# Proinflammatory Phenotype and Increased Caveolin-1 in Alveolar Macrophages with Silenced CFTR mRNA

**DOI:** 10.1371/journal.pone.0011004

**Published:** 2010-06-08

**Authors:** Yaqin Xu, Anja Krause, Hiroko Hamai, Ben-Gary Harvey, Tilla S. Worgall, Stefan Worgall

**Affiliations:** 1 Department of Pediatrics, Weill Cornell Medical College, New York, New York, United States of America; 2 Department of Genetic Medicine, Weill Cornell Medical College, New York, New York, United States of America; 3 Department of Pathology, Columbia University, New York, New York, United States of America; Ludwig-Maximilians-Universität München, Germany

## Abstract

The inflammatory milieu in the respiratory tract in cystic fibrosis (CF) has been linked to the defective expression of the cystic transmembrane regulator (CFTR) in epithelial cells. Alveolar macrophages (AM), important contibutors to inflammatory responses in the lung, also express CFTR. The present study analyzes the phenotype of human AM with silenced CFTR. Expression of CFTR mRNA and the immature form of the CFTR protein decreased 100-fold and 5.2-fold, respectively, in AM transfected with a CFTR specific siRNA (CFTR-siRNA) compared to controls. Reduction of CFTR expression in AM resulted in increased secretion of IL-8, increased phosphorylation of NF-κB, a positive regulator of IL-8 expression, and decreased expression of IκB-α, the inhibitory protein of NF-κB activation. AM with silenced CFTR expression also showed increased apoptosis. We hypothesized that caveolin-1 (Cav1), a membrane protein that is co-localized with CFTR in lipid rafts and that is related to inflammation and apoptosis in macrophages, may be affected by decreased CFTR expression. Messenger RNA and protein levels of Cav1 were increased in AM with silenced CFTR. Expression and transcriptional activity of sterol regulatory element binding protein (SREBP), a negative transcriptional regulator of Cav1, was decreased in AM with silenced CFTR, but total and free cholesterol mass did not change. These findings indicate that silencing of CFTR in human AM results in an inflammatory phenotype and apoptosis, which is associated to SREBP-mediated regulation of Cav1.

## Introduction

CF lung disease is characterized by exaggerated inflammation even in the absence of detectable pathogens [1]. Studies related to inflammation in CF have mostly focused on defective CFTR in lung epithelial cells [2], but CFTR may also play an important role in immune cells [3]–[12].

Alveolar macrophages (AM) serve as first line defense within the respiratory tract, stimulate inflammation and recruit other cells of the immune system [13]. It is not known if AM play a primary role in CF lung disease. Increased numbers of AM were observed in the CF fetal airways [14] and recently in infants with CF [15], suggesting an involvement of AM in the early onset of inflammation. Studies in CF knockout mice suggested a role for CFTR in AM phagosomes and indicated that AM contribute directly to the exaggerated inflammatory response [16], [17]. Impaired clearance of apoptotic cells [18], [19], decreased antigen presentation, and T-cell stimulatory activity [20] have been described in CF lung disease, that could suggest potential functional abnormalities of AM in CF. However, studying the role of CFTR in AM derived from CF lungs is challenging as it is difficult to distinguish if the AM phenotype is primarily induced by the defective expression of CFTR in the AM or induced by the inflammatory milieu resulting from defective CFTR expression in epithelial or other cells [18], [19].

The enhanced inflammatory response in CF has been linked to apoptosis, but the exact mechanisms have been unclear and the results have been contradicting. Increased apoptosis was described in tracheal and pancreatic CF cells [21]–[23]. This was accompanied by an increase in inflammatory cytokines and NF-κB activation, which suggested a common pathway for apoptosis and inflammation in these cells. In contrast, a number of studies relate CFTR expression to apopotosis [24]–[29]. These have linked the lack of CFTR expression or expression of mutant CFTR in CF to a proinflammatory and antiapoptotic phenotype [24]–[29]. Others did not see differences in apoptosis in airway epithelial cells [30]. Furthermore, defective clearance of apoptotic cells in the CF airways was reported to be factor to further trigger the inflammation [18], [19]. The apparent inconsistencies of these findings could be related to the cell-type and apoptosis of AM in CF could play a role in the inflammatory response.

Both, inflammation and apoptosis in macrophages, are associated with caveolin 1 (Cav1) [31]–[33], a membrane protein that has been reported to colocalize with CFTR in epithelial cells [34]. Colocalization of CFTR and Cav1 has been proposed to constitute an “internalization platform”, necessary for appropriate immune response to infection [34]. Cav1 could be a macrophage-specific link between apoptosis and inflammation in CF. The regulation of Cav1 expression is through sterol regulatory element binding proteins (SREBPs), key transcription factors of cellular lipid homeostasis [35]. SREBP expression is primarily regulated by cellular cholesterol [36]. This relevant for CF as CFTR dysfunction has been shown to affect cellular cholesterol and SREBP [37]–[41]. Furthermore, alterations in cellular cholesterol may play a role in the inflammatory phenotype in CF [37], [40].

The goal of this study was (1) to analyze if decreased CFTR expression in human AM affects inflammation and apoptosis by using unstimulated AM derived from non-CF subjects with silenced CFTR expression; (2) to focus on Cav 1 and its regulation by SREBP as a potential factor. We found that silencing of CFTR induced an inflammatory phenotype and augmented apoptosis that were at least partially regulated by SREBP-mediated Cav1 expression. These findings suggest that defective CFTR in AM is relevant for the inflammation in CF lung disease.

## Materials and Methods

### Ethics Statement

Bronchoalveolar lavage (BAL) was performed in 42 clinical healthy volunteers (average 48±12 years, age range 24–79, 31 males and 11 females). Informed consent in written form was obtained from all volunteers according to Institutional Review Board (IRB) guidelines and was approved by the ethics committee of Weill Cornell Medical College (IRB protocol number: 0005004439).

### Cells

Human alveolar macrophages (AM) were obtained by BAL [42] and were processed and cultured as described [13]. Briefly, the lavage fluid was filtered through one layer of gauze, centrifuged (400 g, 10 min) and washed three times in PBS, pH 7.4 (Invitrogen, Carlsbad, CA). Cells were then suspended in full RPMI 1640 medium containing 10% fetal bovine serum, 50 U/ml penicillin, 50 U/ml streptomycin and 2 mM glutamine (Invitrogen) and plated. Macrophage content (always >90%) was determined by Giemsa stain on cytospin preparations (Block Scientific, Inc., Bohemia, NY). Cell viability (always >90%) was determined by trypan blue exclusion. The cells were washed after 3 h to remove non-adherent cells.

### CFTR Knockdown in AM

Five siRNAs specific pre-designed for CFTR were purchased (AM16708A, #1 ID: 145572, #2 ID: 104325, #3 ID: 104323, #4 ID: 4110, #5 ID: 3920; Applied Biosystems, Foster city, CA). To evaluate the siRNA transfection and knockdown efficacy, AM were transfected with different doses of pre-designed GAPDH gene specific siRNA (AM4390850, Applied Biosystems) in siPort as transfection reagent (Applied Biosystems). Transfection with a scrambled siRNA (AM4611, Applied Biosystems) was used as control. After the optimization of siRNA transfection, 100 nM pre-designed CFTR gene specific siRNA (#4 ID: 4110) was selected for the transfection to knockdown of CFTR in AM.

The mRNA levels of CFTR in AM transfected with siRNAs were measured by real-time RT-PCR. RNA was extracted after 48 h from AM transfected with CFTR-siRNA or control-siRNA using TRIzol (Invitrogen). Following reverse transcription of 2 µg RNA, CFTR mRNA was amplified by real-time RT-PCR using a CFTR specific probe (Hs00357011_m1, Applied Biosystems). CFTR mRNA levels were quantified using the ΔΔCt method (Applied Biosystems) and normalized relative to 18s ribosomal RNA (Applied Biosystems). PCR reactions for CFTR and 18s ribosomal RNA were optimized to yield equal amplification efficiency. CFTR protein expression was determined by Western analysis. Total cellular fractions were prepared from AM transfected CFTR-siRNA and control-siRNA after 48 h. Following determination of protein concentration (Micro BCA™ Protein Assay Kit; PIERCE, IL), 100 µg protein was separated by electrophoresis on 4–12% Bis-Tris Gel (NuPAGE@Novex, Invitrogen), transferred to a polyvinylidene difluoride (PVDF) membrane (Bio-Rad Laboratories, Hercules, CA) and incubated with a mouse anti-CFTR antibody (1∶1000, R&D System, Minneapolis, MN). A horseradish peroxidase-conjugated goat anti-mouse secondary antibody (1∶10000, Bio-Rad Laboratories) and Amersham ECL Plus Western Blotting System (GE Healthcare Bio-Sciences, Piscataway, NJ) were used for detection. Following stripping of the membrane, anti-β-tubulin antibody (1∶1000, Sigma-Aldrich, St. Louis, MO) was added to detect β-tubulin as endogenous control. Expression levels of CFTR relative to β-tubulin were quantified using Image J software [43].

### Inflammatory Response

To evaluate if decreased expression of CFTR results in a pro-inflammatory phenotype, we analyzed secreted IL-8 levels and expression of NF-κB in AM transfected with CFTR-siRNA. IL-8 was determined in the culture medium at 0, 2, 4, 8, 24, 48, and 72 h after transfection by ELISA (R&D System), and three measurements were made for each time point. Protein expressions of phosphorylated NF-κB and IκB-α were quantified with CFTR knockdown after 48 h by Western analysis. Fifty µg protein was separated by electrophoresis on 4–12% Bis-Tris Gel (NuPAGE@Novex, Invitrogen), transferred to a PVDF membrane (Bio-Rad Laboratories) and incubated respectively with a mouse anti-phospho-NF-κB p65 antibody (1∶1000, Cell Signaling Technology, Inc., Boston, MA) and with a mouse anti-IκB-α antibody (1∶1000, Cell Signaling Technology, Inc.), and normalized to β-tubulin expression as outlined above.

### Apoptosis

To analyze if AM with decreased CFTR are more susceptible to apoptosis, apoptosis was evaluated by situ terminal deoxynucleotidyltransferase (TUNEL) assay and cleavage of poly (ADP-ribose) polymerase (PARP). AM, plated on coverslip dishes at a density 5×10^5^/well were transfected with CFTR-siRNA or control-siRNA. Cells were fixed with 4% paraformaldehyde (Sigma-Aldrich) after 48 h for 15 min, permeabilized with 0.2% Triton X-100 (Sigma-Aldrich) for 5 min and DNA strand breaks were detected using flurorescein thiocyanate conjugated dUTP. Nuclei were counterstained with 4′-6-Diamidino-2-phenylindole (DAPI) (Sigma-Aldrich). The number of apoptotic cells was determined by fluorescent microscopy (Nikon Instruments, NY) by counting 10 fields. Cleaved PARP protein expression was determined in the cell lysates by Western analysis. Fifty µg protein was separated by electrophoresis on 4–12% Bis-Tris Gel (NuPAGE@Novex, Invitrogen), transferred to a PVDF membrane (Bio-Rad Laboratories) and incubated with a rabbit anti-PARP antibody (1∶200, Santa Cruz Biotechnology, Inc.) and normalized to β-tubulin expression as outlined above.

### Cav1 Expression

To analyze if Cav1, a membrane lipid raft protein, that has been postulated to be colocalized with CFTR in epithelial cells and that is related to inflammation and apoptosis in macrophages, is affected by decreased CFTR expression in AM, we evaluated Cav1 RNA and protein levels. AM were transfected by CFTR-siRNA or control-siRNA and protein and RNA extracted after 48 h as described above. The mRNA of Cav1 was amplified by real-time RT-PCR using a Cav1 specific probe (Hs00184697_m1, Applied Biosystems). Cav1 protein levels were measured by Western analysis. Fifty µg protein was separated by electrophoresis on 4–12% Bis-Tris Gel (NuPAGE@Novex, Invitrogen), transferred to a PVDF membrane (Bio-Rad Laboratories) and incubated with a rabbit anti-Cav1 antibody (1∶200, Santa Cruz Biotechnology, Inc.) and normalized to β-tubulin expression as outlined above.

### SREBP Expression and Activity

As Cav1 is related to lipid metabolism in macrophages, and the SREBP is known to be a major regulator of Cav1 expression, we evaluated expression and activity of SREBP in the AM with CFTR knockdown. Protein expression of SREBP was assessed in AM transfected with CFTR-siRNA or control-siRNA by Western analysis. Fifty µg of total cellular protein was loaded on 4–12% Bis-Tris Gel (NuPAGE@Novex, Invitrogen), transferred to a PVDF membrane (Bio-Rad Laboratories) membrane and probed with rabbit anti-SREBP-1 antibody (1∶1000, BD-Pharmingen, San Diego, CA). Transcriptional activity of SRE was assessed using an adenovirus vector expressing the SRE-promoter of HMG-CoA synthase linked to a luciferase reporter gene and an unregulated nuclear β-galactosidase gene (AdZ-SRE-luc), used to control for transfection efficiency. AM were transfected with CFTR-siRNA and control-siRNA for 48 h, and then infected with AdZ-SRE-Luc at 10^4^ pu/cell for 48 h. Luciferase and β-galactosidase activities were analyzed in the cell lysates by luminometric luciferase and β-galactosidase assays (both, Stratagene, La Jolla, CA). Luciferase was quantified in a luminometer (BD-Pharmingen). The β-galactosidase levels were determined in a microplate luminometer (Bio-Rad Laboratories). Data are expressed as luciferase activity (RLU) normalized to β-galactosidase activity.

### Quantification of Cellular Cholesterol Levels

AM were transfected with CFTR-siRNA and control-siRNA for 48 h. Cell lysates of 5×10^5^ cells were harvested in PBS. Analysis of free and total cholesterol was carried out with 2 injections per sample on a DB17 (0.53-mm ID x 15 mx1 µM) gas-chromatography column at 250°C installed in a Hewlett-Packard 5890 gas-chromatograph equipped with a flame ionization detector (Global Medical Instrumentation Inc., St. Paul, MN). Lipids were extracted with hexane:isopropanol (3∶2, v/v) containing β-sitosterol (5 µg/sample) as an internal standard (Sigma-Aldrich). One third of the extract was used for determination of free cholesterol. The aliquot was dried down, resuspended in hexane (50 µl) and injected into the gas chromatograph. Two thirds of the extract were used to determine total cholesterol after alkaline hydrolysis. To each sample 200 µl KOH (50%, w/v) and 3 ml methanol were added, the mixture was capped with argon, vortexed and incubated at 80°C for 1 h, followed by lipid extraction using 3 ml of water and 5 ml of HPLC grade hexane (Sigma-Aldrich). Following centrifugation at 500 g for 5 min the hexane phase was concentrated by evaporation, resuspended in hexane and injected into the gas chromatograph. The difference between total cholesterol and free cholesterol is esterified cholesterol, and the mass of cholesterol ester is calculated as 1.67 x esterified cholesterol.

### Statistics

The results are presented as mean ± standard error of the mean (SEM). Significance was calculated using the student t-test.

## Results

### CFTR Knockdown in AM

**Figure 1:**
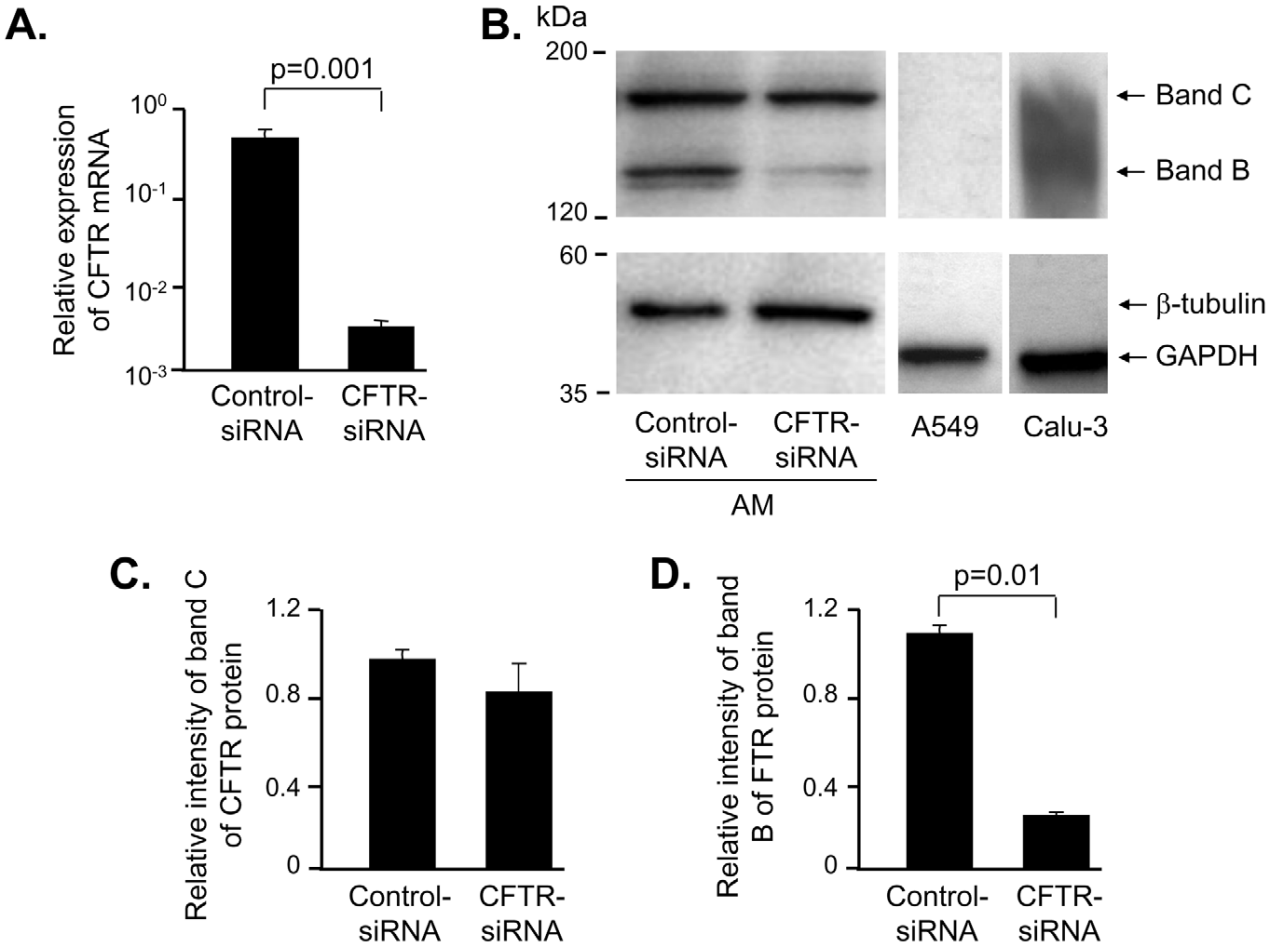
CFTR knockdown in AM. Human AM, obtained by bronchoalveolar lavage from healthy adults, were transfected with CFTR-siRNA or control-siRNA and analyzed after 48 h for CFTR mRNA and protein by real-time RT-PCR and Western analysis. To confirm the specificity of CFTR antibody, A549 cells, which do not have intrinsic CFTR expression, and CFTR expressing cell line Calu-3 cells were used as negative and positive controls in the Western analysis. **A.** Real-time RT-PCR. Human 18s ribosomal RNA was used as normalization control. **B.** Western analysis. B-tubulin or GAPDH was used as control. CFTR protein was detected as an mmature form (band B) at the size of 150 kDa and a mature form (band C) at the size of 170 kDa. **C.** and **D.** Quantification of Western analysis. Shown is the mean ± SEM of three pairs of independent samples. This experiment is the representative of 6 studies.

Efficiency of CFTR knockdown in human AM following transfection with CFTR specific siRNA (CFTR-siRNA) was assessed by CFTR mRNA and protein expression. The siRNA transfection efficiency in AM could be achieved 95% with the transfection of GAPDH-siRNA labeled with green fluorescence and knockdown of GAPDH gene measured by real-time PCR was more than 90% compared to the scrambled control siRNA (control-siRNA) (data not shown). Forty eight hours after transfection, CFTR mRNA levels were reduced more than 100-fold in CFTR-siRNA treated cells compared to the cells transfected with control-siRNA (p = 0.001; Figure 1A). CFTR protein is known to have an immature form (band B) with the size of 150 kDa and a fully glycosylated mature form (band C) with the size of 170 kDa. Our data showed that significant decrease of immature band B of CFTR protein while the mature form band C remained unchanged after 48 h CFTR-siRNA transfection (Figure 1B). The quantification of CFTR protein showed no decrease of band C (Figure 1C) and a 5.2-fold reduction of band B (p = 0.01; Figure 1D) in AM transfected with CFTR-siRNA compared to the cells transfected with control-siRNA. The similar size of CFTR protein was observed in the positive control Calu-3 cell lysate and there was no band in negative control A549 cell lysate, confirming the specificity of the CFTR antibody (Figure 1B).

### Increased Inflammatory Response in AM with CFTR Knockdown

**Figure 2:**
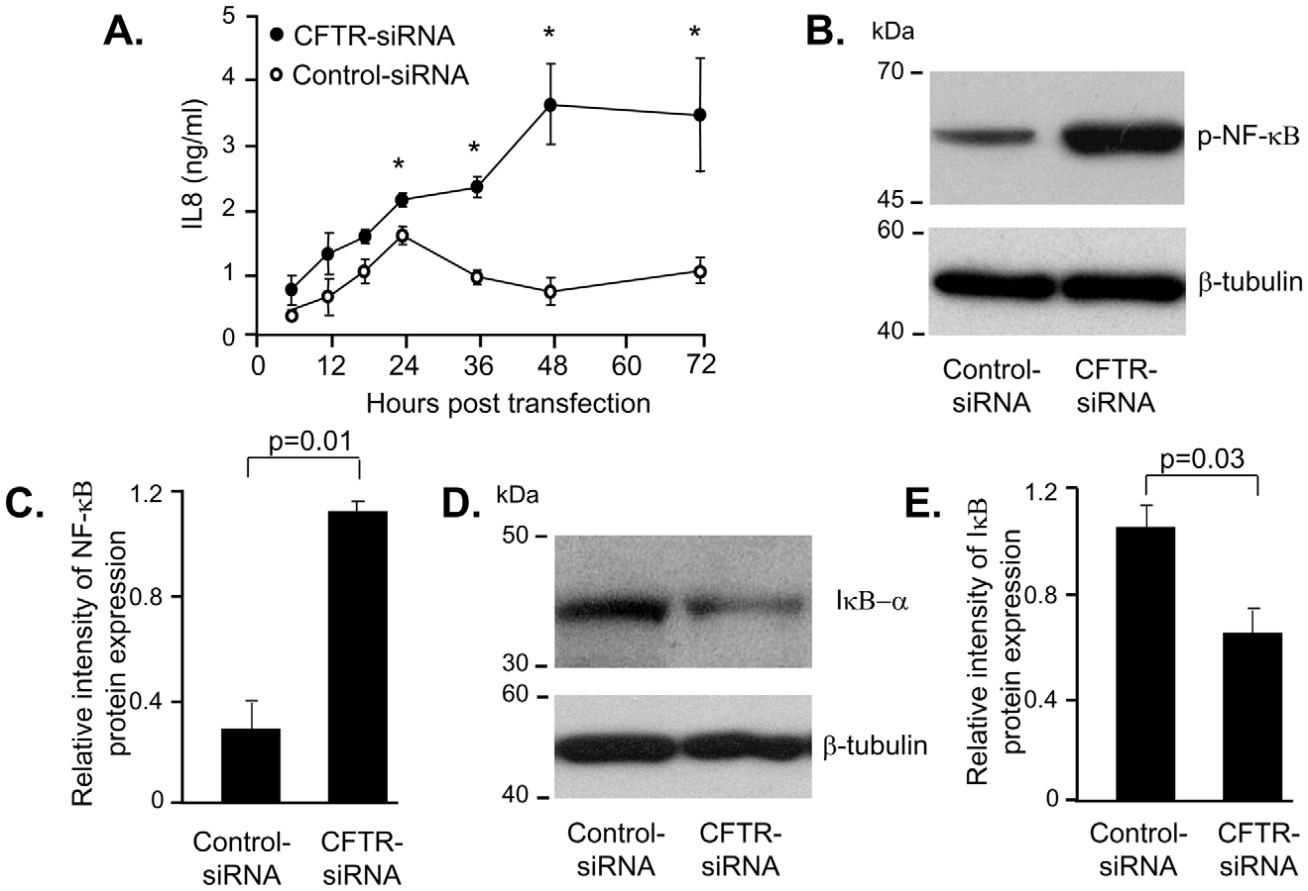
Increased IL-8 secretion of AM with CFTR knockdown. **A.** AM, transfected with CFTR-siRNA or control-siRNA, were analyzed after 6, 12, 18, 24, 36, 48, and 72 h for IL-8 in the cell culture supernatant by ELISA (* denotes p value: p = 0.03 at 24 h; p = 0.01 at 36 h; p = 0.001 at 48 h; p = 0.004 at 72 h). **B.** AM, transfected with CFTR-siRNA or control-siRNA after 48 h, were evaluated for phosphorylated NF-κB protein expression of by Western analysis. **C.** Quantification of phosphorylated NF-κB Western analysis. **D.** AM, transfected with CFTR-siRNA or control-siRNA after 48 h, were also evaluated for IκB-α protein expression of by Western analysis. **E.** Quantification of IκB-αWestern analysis. Shown is the mean ± SEM of three pairs of independent samples. This experiment is the representative of 6 studies.

To analyze if CFTR silencing in human AM affects inflammatory cytokine secretions, IL-8 levels were analyzed in cell culture supernatants. Compared to human AM transfected with control-siRNA, IL-8 levels were increased in the supernatant obtained from human AM transfected with CFTR-siRNA at 24, 36, 48, and 72 h post transfection (p = 0.03 at 24 h; p = 0.01 at 36 h; p = 0.001 at 48 h; p = 0.004 at 72 h; Figure 2A). As NF-κB is known to be a positive regulator of IL-8 secretion we analyzed phosphorylated NF-κB and IκB-α, one of three inhibitory proteins of NF-κB activation in the AM with silenced CFTR. Forty eight hours after transfection, phosphorylated NF-κB, detected at a size of 65 kDa (Figure 2B), was increased 3.7-fold, as estimated by densitometry quantification (p = 0.01) in human AM transfected with CFTR-siRNA (Figure 2C). Expression of IκB-α (Figure 2D) was decreased 1.7-fold (p = 0.03) in human AM with silenced CFTR (Figure 2E). The data suggest that reduced production of CFTR induces a proinflammatory phenotype in human AM.

### Apoptosis in AM with CFTR Knockdown

**Figure 3:**
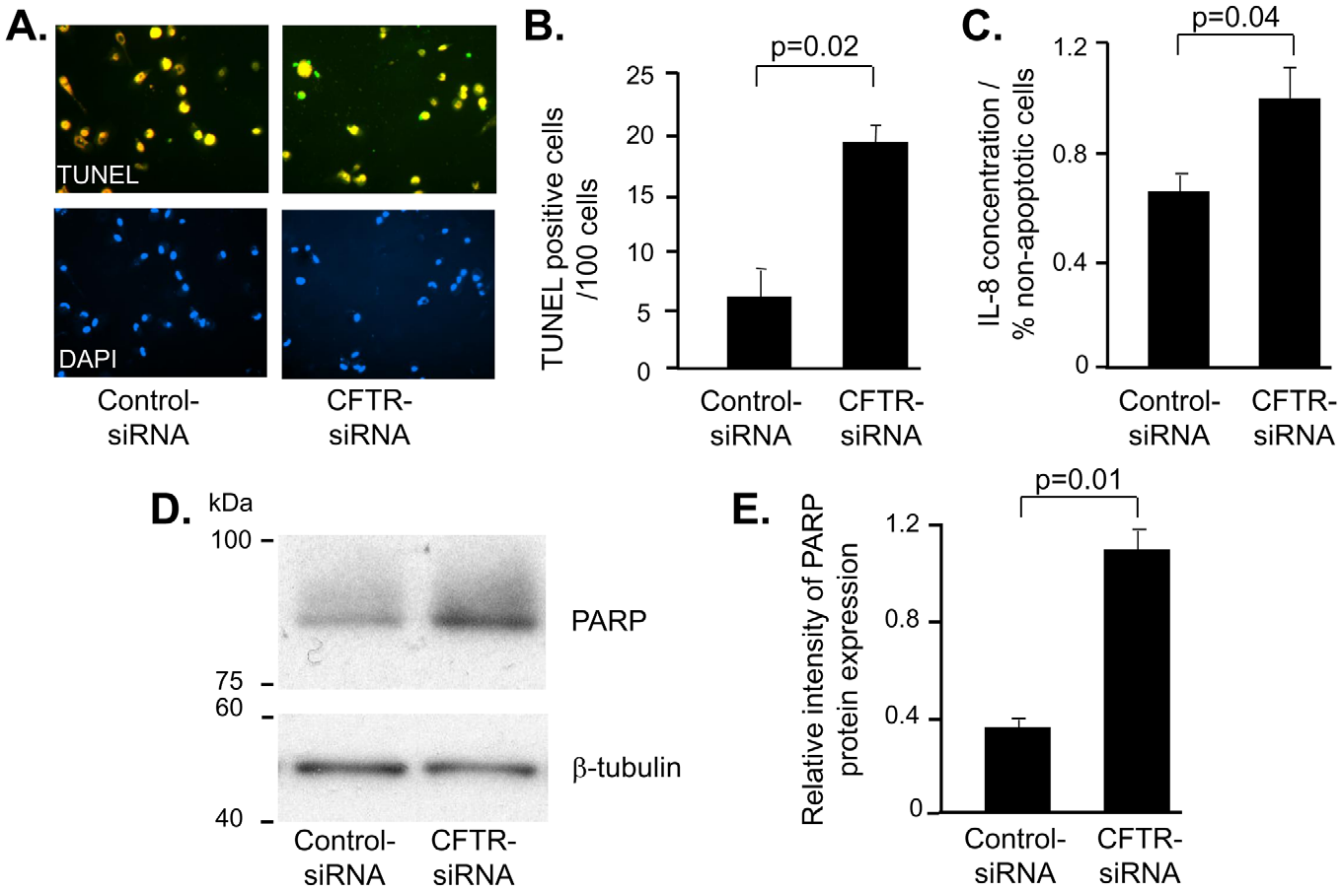
Increased apoptosis in AM with deficient CFTR. AM transfected with CFTR-siRNA or control-siRNA for 48 h were analyzed for apoptosis by TUNEL assay and for cleaved PARP protein expression by Western analysis. **A.** TUNEL assay. The nuclear staining of green fluorescence was shown as the positive apoptosis signal. DAPI served as normal nuclear staining control. **B.** Quantification of TUNEL assay. **C.** IL-8 secretion adjusted to the percentage of non-apoptotic cells in AM transfected with CFTR-siRNA or control-siRNA after 48 h. **D.** Western analysis of cleaved PARP protein expression using β-tubulin as loading control. **E.** Quantification of Western analysis. Shown is the mean ± SEM of three pairs of independent samples. This experiment is the representative of 6 studies.

To assess if the inflammatory phenotype of the AM with silenced CFTR would affect apoptosis, apoptosis was evaluated by TUNEL assay and evaluation of caspase-3 mediated cleavage of poly (ADP-ribose) polymerase (PARP) in human AM 48 h after transfection with CFTR-siRNA or control-siRNA. Apoptotic cells were detected by TUNEL assay in AM transfected CFTR-siRNA (visualized as yellow fluorescence superimposed by blue fluorescence DAPI nuclear staining and green TUNEL staining; Figure 3A) and were increased compared to AM transfected with control-siRNA (p = 0.02; Figure 3B). To correlate the apoptosis with the proinflammatory phenotype, the proportion of IL-8 secretion to the percentage of non-apoptotic cells was measured in AM transfected with CFTR-siRNA or control-siRNA. At 48 h, higher IL-8 production was detected in AM with CFTR knockdown compared to the control (p = 0.04; Figure 3C). The expression of PARP, a well known apoptosis indicator, was evaluated by Western analysis. The cleaved form of PARP (89 kDa) was increased in human AM tranfected with CFTR-siRNA (Figure 3D). Quantification of PARP protein by densitometry revealed a 3.3-fold increase in human AM transfected with CFTR-siRNA compared to controls (p = 0.01; Figure 3E). The data suggest that knockdown of CFTR promotes apoptosis in human AM.

### Cav1 is Increased in AM with CFTR Knockdown

**Figure 4:**
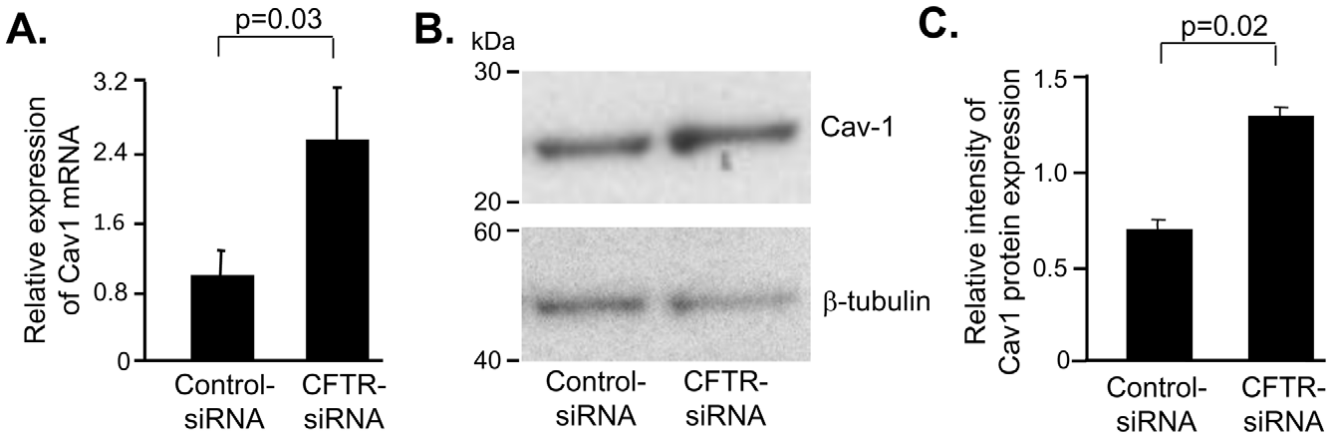
Cav1 expression is increased in AM with deficient CFTR. AM transfected with CFTR-siRNA or control-siRNA were analyzed for Cav1 mRNA and protein expression after 48 h. **A.** Real-time RT-PCR. The human 18 s ribosome RNA served as the normalization control. **B.** Western analysis. B-tubulin was used as control. **C.** Quantification of Western analysis. Shown is the mean ± SEM of three pairs of independent samples. This experiment is the representative of 6 studies.

We next evalaluated the expression of Cav1, a component of cellular membrane lipid rafts, that colocalizes with CFTR and is involved in regulation of inflammatory responses and apoptosis in macrophages. Forty eight hours after transfection Cav1 mRNA was increased 2.7-fold (p = 0.03) in human AM transfected with CFTR-siRNA compared to controls transfected with control-siRNA (Figure 4A). Consistently, protein expression of Cav1, detected at size of 22 kDa, was increased in human AM tranfected with CFTR-siRNA compared to controls (Figure 4B). Quantification of Cav1 protein expression by densitometry revealed a 2.2-fold (p = 0.02) increase in human AM transfected with CFTR-siRNA compared to controls (Figure 4C).

### SREBP Cleavage and SRE Transcription Activity in AM with CFTR Knockdown

**Figure 5:**
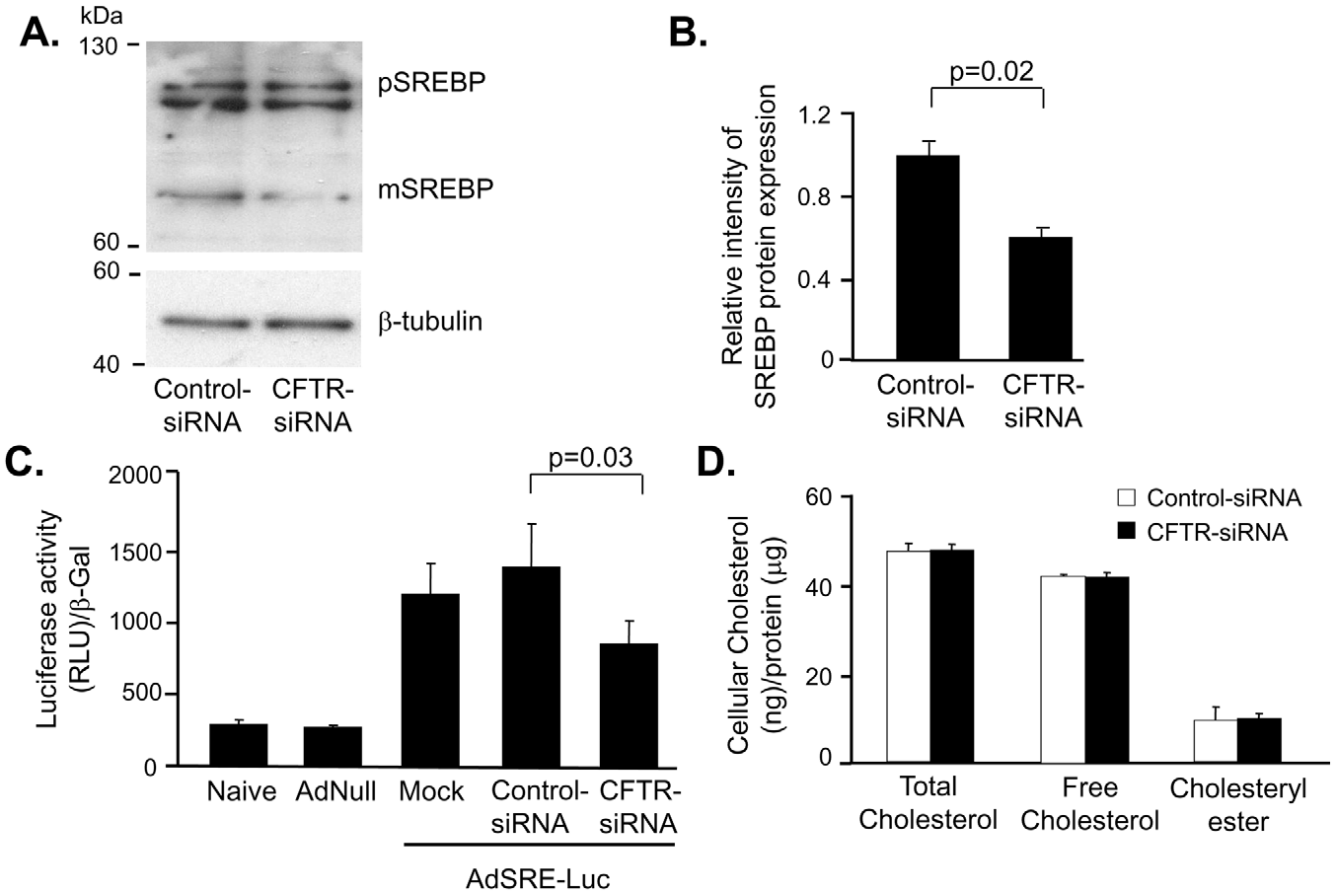
Decreased cleavage of the SREBP and transcription activity of SRE in AM with deficient CFTR. **A.** AM transfected with CFTR-siRNA or control-siRNA were evaluated after 48 h for SREBP protein expression by Western analysis. **B.** Quantification of Western analysis. **C.** Transcription activity assay of SRE. AM, transfected with CFTR-siRNA for 48 h, were infected with AdZ-SRE-luc. The transcriptional activity of SRE was measured by luciferase assay using β-gal as normalization control. **D.** Total cellular cholesterol, free cholesterol, and cholesterol ester were measured by liquid chromatography. Shown is the mean ± SEM of three pairs of independent samples. This experiment is the representative of 6 studies.

We next analyzed if the increased Cav1 expression could have been regulated by SREBP, a known negative regulator of its transcription and that has been found to be increased in CF epithelial cells. Expression and activity levels of SREBP in AM with CFTR knockdown were evaluated by Western analysis and promoter reporter gene expression. Western analysis, carried out 48 h after transfection, revealed decreased expression of mature SREBP (mSREBP) (68 kDa), the transcriptionally active protein that is cleaved, by a multi-step process, from the inactive precursor from, precursor SREBP (pSREBP) (125 kDa) in human AM transfected with CFTR-siRNA compared to human AM transfected with control-siRNA (Figure 5A). Concentration of mSREBP was decreased 2-fold in AM with silenced CFTR (p = 0.02; Figure 5B) as estimated by densitometry. To evaluate SRE-mediated gene transcription, human AM were transduced with AdZ-SRE-luc, an Ad vector expressing a luciferase reporter gene under the control of an SRE-promoter or AdNull, a control vector. Consistent with decreased mSREBP levels, transcriptional activity of SRE was reduced in CFTR-deficient human AM compared to human AM transfected with control-siRNA (p = 0.03; Figure 5C).

SREBP is a key transcription factor of cellular lipid homeostasis and is usually regulated by altered cellular cholesterol. An altered cellular cholesterol found in CF epithelial cells has been proposed to lead to SREBP activation, we determined cellular cholesterol levels in the AM with silenced CFTR. Total and free cholesterol mass were analyzed by gas chromatography and determined cholesteryl ester mass by substraction of free cholesterol from total cholesterol. Total cholesterol and free cholesterol mass were not different in human AM transfected with CFTR-siRNA or control-siRNA 48 h or 63 h (data not shown) after transfection (Figure 5D). We measured de novo synthesis of free cholesterol by evaluating incorporation of ^3^H-mevalonolactone into free cholesterol over 16 h and incorporation of ^3^H-oleate into cholesteryl-ester over 4 h and found no difference between both experimental groups (data not shown). The finding of unaffected cholesterol mass in the presence of increased Cav1 expression and decreased mSREBP are consistent with a model in which knockdown of CFTR in human AM induces a redistribution of regulatory pools of cholesterol.

## Discussion

The contribution of AM to the hyperinflammatory milieu in the CF lung is not clear. The present study attempts to address this question by analyzing AM from healthy donors in which expression of CFTR is decreased by siRNA-mediated knockdown. AM with decreased CFTR expression exhibited an inflammatory phenotype as evidence by increased IL-8 and NF-κB, similar to the phenotype described in epithelial cells that express no or defective CFTR [44]–[46]. In contrast to data obtained in epithelial cells, our results demonstrated an inverse relationship between expression of CFTR and apoptosis as well as expression of mSREBP [39]. The data suggest an AM specific phenotype in which increased expression of Cav1 could be a consequence of decreased mSREBP and cause for increased apoptosis in this cell type.

A hyperinflammatory response is a hallmark of CF lung disease [47]. CFTR-deficient epithelial cells or epithelial cells treated with CFTR chloride channel inhibitors show increased secretion of IL-8 [44], [46]. Elevated levels of proinflammatory cytokines, such as IL-8 and IL-6, and decreased levels of the anti-inflammatory cytokine IL-10 are characteristic findings in the bronchioalveolar lavage fluid of CF patients even in the absence of pathogens [1], [48], [49]. These cytokines are thought to be primarily produced by the CFTR-deficient epithelial cells, however increased baseline levels of IL-8 have also been observed in blood monocytes of CF patients [12] and in AM derived from CF knockout mice [17]. Our results that demonstrate increased secretion of IL-8 and phosphorylated NF-κB, a positive regulator of IL-8 expression in human AM obtained from healthy donor in which CFTR expression was decreased by siRNA-medaited knockdown are consistent with this shifting paradigm [50]. Increased activation of NF-κB in CFTR-deficient epithelial cells has been thought to partially be a result of abnormal trafficking of mutant CFTR protein CFTRΔF508, where its accumulation in the ER induces intracellular stress resulting in NF-κB activation [45]. Our findings suggest that the lack of CFTR in AM alone triggers the hyperinflammatory response and the activation of NF-κB in AM and emphasize the potential significance of this cell type in CF lung disease.

Although expression of CFTR in human AM is relatively low compared to epithelial cells [11], knockdown by transfection of CFTR specific siRNA significantly decreased protein expression. As the half-life of plasma membrane CFTR exceeds 48 h [51], we did not observe a decrease of the mature form of CFTR. Other studies using CFTR siRNA also demonstrated the decrease of single band of CFTR protein [38], [52]–[54], which presumably also reflects the 150 kDa immature form. Because we could not keep AM with silenced CFTR in culture for extended periods to see decreased expression of the mature form of the protein, the observed effects could reflect the effect of intracellular trafficking rather than reduced CFTR expression on the cell membrane. In addition, our model using silencing of CFTR expression for short term culture may not reflect long term phenotypes of AM in the lower airways in the CF lung and also does not predict the phenotype induced by CFTR trafficking mutations such as ΔF508.

The relationship between CFTR and apoptosis is currently not clear. Several studies that investigated susceptibility to apoptosis in cell lines and tissue obtained from CF patients have yielded inconclusive results [21]–[30]. On one hand, increased apoptosis was observed in small intestine biopsies from CF patients [23]. Pancreatic apoptosis, associated with over-expression of IL-8 and activation of NF-κB pathway, was proposed as a possible mechanism for CF-related diabetes [21], [22]. Other studies however, did not observe increased baseline apoptosis in respiratory epithelial cells expressing mutant CFTR except after exposure of the cells to *P. aeruginosa* [30].

In contrast, several other studies reported that expression of defective CFTR or knockdown of CFTR protects against apoptosis phenotype [24]–[29]. CF knockout mice exhibited a baseline proinflammatory state and an anti-apoptotic phenotype in the pancreas [24] and showed failed induction of apoptosis in response to *P. aeruginosa* [28]. Recently, delayed apoptosis has been described in polymorphonuclear neutrophil (PMN) from CF patients, which might explain PMN persistence in the CF lung [29].

Our study showed increased baseline apoptosis in AM with CFTR knockdown. The increased apoptosis of inflammatory cells such as macrophages and neutrophils has been considered as an anti-inflammatory phenotype, however our study showed both augmented apoptosis and increased proinflammatory in AM with CFTR knockdown. Altered apoptosis has not been described for disruption of other epithelial chloride channels, including members of volume-regulated chloride channel CLC family [55], [56]. We suggested that as a consequence of more proinflammatory cytokine production the cells are prone to undergo apoptosis. Consistent with our findings, increased Cav1 has been observed in murine peritoneal macrophages undergoing apoptosis [31]. These contradictory observations could be related to cell type-specific responses and/or the agents used to induce apoptosis. Again, as outlined above, the phenotype of AM in the CF lung may not be accurately predicted by the short term culture results of our study.

Defective or absent CFTR is known to be associated with abnormalities in the cellular lipid metabolism [40], [57], [58]. The mSREBP and membrane free cholesterol levels have been shown to be increased in epithelial cells that express defective CFTR [39]. It is, however not clear whether these changes in cholesterol metabolism are cell-type or CFTR mutation specific, i.e. only found in cells that express ΔF508 CFTR protein [37]. Notably, knockdown of CFTR in human AM did not affect total and free cellular cholesterol levels in our study. On one hand, the lack of difference in cholesterol mass could be related to differences in cell type. In epithelial cells, cholesterol is mainly found in the plasma membrane, in macrophages, a significant amount of cholesterol can be found stored in intracellular compartments. Notably, failure to detect differences in total and free cholesterol mass does not exclude increased free cholesterol in the plasma membrane, but is rather supported by the finding of increased Cav1 mass, a protein known to bind free cholesterol in caveolae.

Increased expression of Cav1 in the present study following decrease of CFTR expression in AM supports that CFTR and Cav1 interact in this cell type. Potential explanation for increased SREBP in CF epithelial cells and decreased SREBP in AM macrophages that lack CFTR could be that intracellular cholesterol pools to the decreased mSREBP expression and decreased SRE activity seen in the AM with decreased CFTR expression, as Cav1 expression is negatively regulated by SRE in macrophages. Furthermore, it could also explicate the unchanged cellular cholesterol levels seen in the present study.

In summary, the present study points to a role in CFTR in AM, that may be similar to what is known for epithelial cells, as decreased CFTR expression resulted in an inflammatory cellular phenotype. In addition, AM with decreased CFTR expression showed augmented apoptosis, increased expression of Cav1, and decreased activation of SRE, which may be specific for this cell type. Lack of CFTR expression in AM may play a role in CF lung disease and the study of the abnormalities associated with lack of CFTR expression in this cell type may aid in understanding the complex cellular functions that CFTR is involved in.
